# Hajj health examination for pilgrims with asthma in Malaysia: An ethnographic study

**DOI:** 10.7189/jogh.12.04023

**Published:** 2022-03-26

**Authors:** Rizawati Ramli, Nik Sherina Hanafi, Norita Hussein, Ping Yein Lee, Sazlina Shariff Ghazali, Ai Theng Cheong, Ahmad Ihsan Abu Bakar, Azah Abdul Samad, Suhazeli Abdullah, Hilary Pinnock, Aziz Sheikh, Ee Ming Khoo, the RESPIRE Collaborators

**Affiliations:** 1 Department of Primary Care Medicine, Faculty of Medicine, Universiti Malaya, Kuala Lumpur, Malaysia; 2 UMeHealth Unit, Faculty of Medicine, Universiti Malaya, Kuala Lumpur, Malaysia; 3 Faculty of Medicine and Health Sciences, Universiti Putra Malaysia, Selangor, Malaysia; 4 Hospital Pusrawi Pvt Ltd, Kuala Lumpur, Malaysia; 5 Ministry of Health, Putrajaya, Malaysia; 6 Usher Institute, University of Edinburgh, Edinburgh, UK

## Abstract

**Background:**

Asthma was one of the top causes of hospitalization and unscheduled medical attendances due to acute exacerbations and its complications. In Malaysia, all pilgrims must undergo a mandatory health examination and certified fit to perform pilgrimage. We studied the current organisational and clinical routines of Hajj health examination in Malaysia with a focus on the delivery of care for pilgrims with asthma.

**Methods:**

We conducted non-participant observation to obtain ethnographic understanding of Hajj health examination activities for 2019. Observations were guided by a checklist and recorded as notes that were analysed thematically. The study was conducted at 11 public (from each region in Malaysia, namely, North, South, East, West of Peninsular Malaysia, and Sabah and Sarawak of East Malaysia) and two private primary care clinics.

**Results:**

We observed considerable variation in the implementation and practice of Hajj health examinations among the 11 public clinics but no marked variation among the private clinics. The short time span of between three to four months was inadequate for disease control measures and had put pressure on health care providers. They mostly viewed the Hajj health examination as merely a certification of fitness to perform the pilgrimage, though respiratory health assessment was often inadequate. The opportunity to optimise the health of pilgrims with asthma by providing the appropriate medications, asthma action plan and asthma education including the preventive measures was disregarded. The preliminary health screening, which aimed to optimise pilgrims’ health before the actual Hajj health examination was not appreciated by either pilgrims or health care providers.

**Conclusions:**

There is great potential to reform the current system of Hajj health certification in order to optimise its potential benefits for pilgrims with asthma. A systematic approach to restructuring the delivery of Hajj health examination could address the time constraints, clinical competency of primary health care providers and resources limitations.

We would like to dedicate this paper to the memory of Prof Dr Liew Su May.

The Hajj pilgrimage is a religious obligation for all adult Muslims who are physically fit and financially able [1]. This congregational act of worship that takes place in the Kingdom of Saudi Arabia is one of the largest human gatherings, with an estimated two to three million pilgrims annually [2]. Globally, 1.6 billion Muslims desire to perform Hajj at least once in their lifetime. Therefore, the Saudi government places country quotas on the number of pilgrims allowed. Before the Covid-19 pandemic, the quota for Malaysia was about 30 000 pilgrims per year. The Hajj Fund Board (HFB) (or its official corporate name Lembaga Tabung Haji), is an Islamic institution in Malaysia that manages fund deposited by Muslims and provides Hajj services for Malaysian pilgrims. It ensures a well-organised and safe Hajj operation to facilitate pilgrims obtaining a ‘Mabroor’ Hajj (Hajj that is accepted by God). Muslims who deposit money in HFB can register as prospective pilgrims and use their fund to perform the Hajj.

The pilgrimage is challenging even for those who are physically fit and able. It involves long walks of between 7-10 km per day for at least five days, performing compulsory rituals over rough terrain in extreme climates, some of which occur in confined spaces [3-6]. The cause of hospitalisation due to respiratory diseases during Hajj was estimated between 13.5% to 57.0% [4,6,7]. Cough, common cold, sore throat and shortness of breath are the commonest respiratory symptoms seen among pilgrims [8-11]. Malaysia has mandated all pilgrims to undergo the Hajj health examination and to be certified fit in order to perform Hajj. Hajj health examination is an activity whereby prospective pilgrims of the given year undergo clinical evaluation by primary care doctors with the aim of optimising pilgrims’ physical and mental health to perform the rituals. This is organised by the HFB with assistance from the Ministry of Health, Malaysia (MOH). Pilgrims are given the option to undertake the examination at any selected public or private primary care clinics in their district. Public clinics are governed and fully funded by the government of Malaysia whilst private clinics are privately owned, funded and operated by a group of or an individual. Public clinics have been catering for the majority of the Hajj health examinations, which are normally conducted over a few days.

**Table 1** shows the organisation of the Hajj health certification in 2019 which outlines the process from the issuance of Hajj offer letter by HFB through departure for pilgrimage. The modules used for the training of doctors cover general physical examination, assessment of daily life activities, renal diseases and psychiatric assessment. HFB had also produced a guideline book for Hajj health examination [12] for health care providers’ further references. The doctors consisted of doctors from public and private primary care clinics. A ‘preliminary health screening’ was instituted in 2019 to identify and resolve medical issues in advance of the Hajj health examination. It is not mandatory but is encouraged especially for pilgrims with chronic disease(s) and can be done by their regular doctors and/or clinics. The Hajj treatment record book (BRRJH) is a record of the pilgrims’ medical history and examination, vaccination, and the status of fitness certification for Hajj. There are also sections for pilgrims’ self-reported medical problems. This book is mandatory for use during the Hajj health examination and has to be carried by pilgrims throughout the pilgrimage.

**Table 1:** Organisation of the Hajj health certification process

**Before Hajj health examination (6-7 months before departure)**	1. Pilgrims receive Hajj offer letter; together with; (i) preliminary health screening form (Figure 1) (ii) Hajj pilgrim’s treatment record book (BRRJH)
	2. Compulsory training of doctors; using standardised modules prepared by the Centre of Disease Control, MOH
	3. Optional courses for pilgrims: 16 educational sessions including one on health
**During Hajj health examination at centralised or individual primary care clinics (within 3-4-month period)**	1. Clinical evaluation and consultation
	2. Outcome of examination; (i) pass, (ii) fail or (iii) refer to family medicine specialist or to specialised disciplines at tertiary centres for further evaluation
	3. Meningococcal vaccination for pilgrims who pass
**After Hajj health examination, at individual primary care clinics (Up to 1 month before departure)**	1. Re-evaluation of referred cases
	2. Outcome of re-evaluation; (i) pass or (ii) fail
	3. Meningococcal vaccination for pilgrims who pass

BRRJH – pilgrims treatment record book, MOH – Ministry of Health

**Figure 1:**
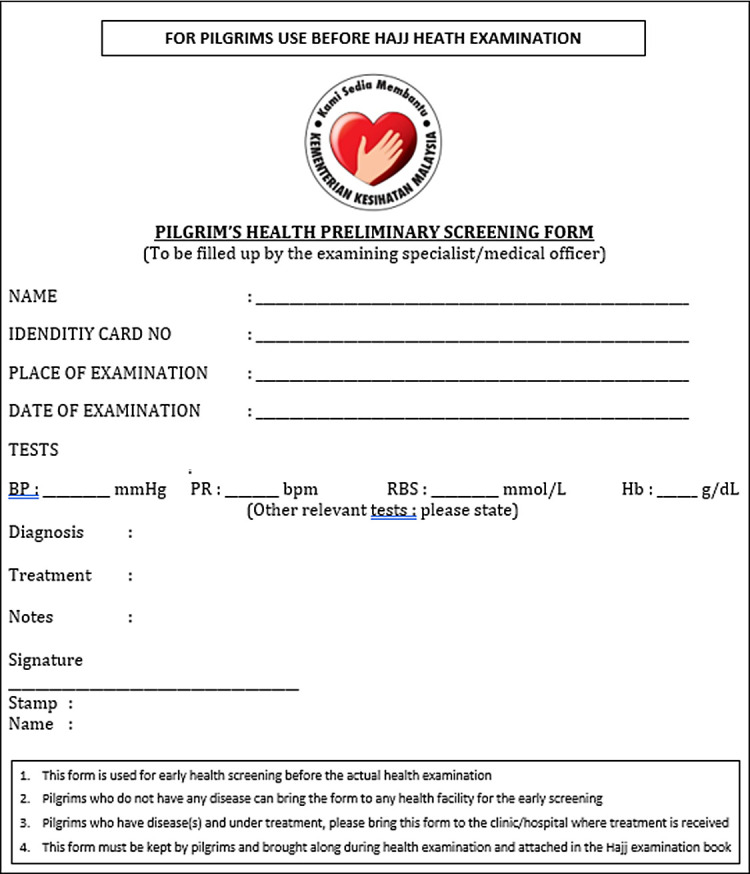
Translated version of preliminary health screening form.

Theoretically the system should ensure that pilgrims with uncontrolled asthma achieve good control before embarking on the pilgrimage [12]. In neighbouring Indonesia, despite the compulsory Hajj health examination, 47% of partly controlled and 61% of uncontrolled pilgrims with asthma at embarkation had exacerbations during Hajj [13]. There is no study to date of its incidence among Malaysian pilgrims. However, a nationwide study across primary care centres in Malaysia found that based on GINA guidelines on asthma control, 41% had well-controlled, 38% partly controlled and 21% uncontrolled asthma [14]. Hence, we aimed to observe the implementation of Hajj health examination in Malaysia using asthma as an exemplar chronic disease to inform future strategies to improve care among pilgrims.

## METHODS

### Study design

We conducted non-participant observation to obtain ethnographic understanding [15] of the organisational and clinical routines of the Hajj health examination for pilgrims with asthma in 2019. The study was conducted at 11 public and two private primary care clinics. Two clinics were selected from each region in Malaysia, namely, North, South, East, West of Peninsular Malaysia, and Sabah and Sarawak of East Malaysia. The clinics were selected to represent a range of organisational arrangements in primary care clinics including the location (urban and rural), infrastructure and facilities (large and small clinics) and the estimated number of pilgrims in the district. We obtained consent from the person in-charge of the Hajj health examination in the selected clinics.

### Data collection

The observations were conducted for one whole day for clinics with many pilgrims and two days for clinics with small number of pilgrims. A checklist was used to facilitate the observation of the Hajj health examination processes [16]. The checklist was developed prior to observation through discussions among researchers and was adapted from domains described by Spradly, namely space, actors, activities, objects, actions, occasions, sequence, goals, and feelings [17]. Specific observations included the organisational preparation, appointments arrangement, clinical assessments and outcomes, continuity of care, vaccinations, management of pilgrims who were deemed unfit, access and communication of information to pilgrims.

### Data analysis

Researchers’ observation notes were documented and entered into Microsoft Word documents, which were transferred into the NVivo 12 software for coding. Two researchers independently coded the first observation notes based on the domain framework. Domain analysis involved systematic identification of components of the observed scene and possible subcategories. The observation notes were coded and categorised. Any discrepancies were discussed, and consensus reached to formulate the coding framework, which was then used to code the rest of the observation notes. Researcher AIB compared all the codes for accuracy and the codes were analysed thematically with the team. All researchers constantly reflected on the possible biases they had that could have influenced the interpretation during observation and analysis.

### Patient and public involvement

Patients and public have helped in developing and shaping the research questions and design of this research.

### Ethics approval

This study received permission from the State Health Department directors and ethics approval from the National Medical Research Register - Medical Research and Ethics Committee (NMRR-MREC) (NMRR-18-2997-43555), from the MOH and from the sponsor: Academic and Clinical Central Office for Research and Development (ACCORD) ethics committee, United Kingdom.

## RESULTS

### Public primary care clinics

We observed variation in the organisation of the Hajj health examination at the 11 public primary care clinics. Two clinics catered pilgrims only from their clinic coverage area (decentralised) while the rest accepted pilgrims from the whole district (centralised). The number of pilgrims examined in each clinic ranged from 7 to 324 per day. There was no fee imposed on the pilgrims.

All appointments for the Hajj health examination had been arranged by the HFB and district health offices, and agreed upon by the clinics. Each clinic varied in their scheduling of examinations depending on the number of pilgrims allotted, the clinic workload and the availability of resources including the human resource, imaging, and laboratory facilities. Some conducted the Hajj examinations over the weekend while others conducted the examinations during weekdays amidst normal clinic activities. All but one clinic only examined pilgrims who had a scheduled appointment. The clinic in exception contacted pilgrims prior to examination date to confirm attendance; anyone who defaulted on the scheduled day were contacted and pilgrims who attended without appointment were also being seen.

All pilgrims were instructed by the HFB to bring all relevant medical documents and medication to facilitate the clinical assessment by doctors. The preliminary health screening form, if done and completed, was used as a guide for doctors. However, for a number of reasons this did not routinely happen. Some pilgrims did not receive the form while some who received, had not undergone this preliminary screening. Some pilgrims who had completed the screening did not bring the form on the examination day, so assessments or tests that had already been done had to be repeated. Some doctors were not aware of or did not check the preliminary screening form. The majority of the pilgrims at centralised clinics were not the clinics’ regular patients and hence, their medical records were not available to the doctors on duty.

Various categories of health care personnel were involved in the Hajj health examination. Most of them were staff from the clinic itself while others were deployed from other clinics or the district health office. The ratio of doctors to pilgrims for the examinations ranged from 1:3 to 1:37. **Table 2** and **Table 3** summarise the overall findings of observations at the 11 public primary care clinics.

**Table 2:** Observations related to organisation of Hajj health examination in public primary care clinics (n = 11)

	Clinic 1	Clinic 2	Clinic 3	Clinic 4	Clinic 5	Clinic 6	Clinic 7	Clinic 8	Clinic 9	Clinic 10	Clinic 11
**Examination time frame**	7 d	1-d	2 d	1 d	Until no more pilgrims came	1 d	2-d	Half working day	Until no more pilgrims came	5 d	1 d
**Examination day**	Weekdays	Saturday	Saturday and Sunday	Saturday	Weekdays	Saturday	Saturday and Sunday	Weekdays	Weekdays	5 consecutive Sundays	Saturday
**Specific time**	8:30 am -10:30 am	8 am – until no more pilgrims came	8 am – 2 pm	8 am–1p.m.	8.30 am – 10.30 am	8 am, 11 am, 2 pm	Half day from 8 am	6 pilgrims/30 min	Amidst normal clinic	9 am -12 pm	Until no more pilgrims attended
**Organisation**	Centralised	Centralised	Centralised	Centralised	Decentralised	Centralised	Centralised	Centralised	Decentralised	Centralised	Centralised
**No. of pilgrims (load)**	Total 118	Total 324	Total 252	Total 100-150	Maximum 20 pilgrims/d	Total 170	Total 100	60 pilgrims/d	Average 7 pilgrims/d	Total 200	Total 280
	20 slots/d		Day 1 – 263, day 2 – 116								
**Staff (same clinic /deployed from other clinics)**	Staff from the organising clinic and deployed from six other clinics.	Staff from the organising clinic and deployed staff from other clinics.	Staff from the organising clinic and deployed staff from other clinics.	Staff from the organising clinic and deployed staff from other clinics.	Staff of the clinic	Staff from the organising clinic and deployed staff from other clinics.	Staff from the organising clinic and deployed staff from other clinics.	Staff from the organising clinic and deployed staff from other clinics.	Staff of the clinic	Staff from the organising clinic and deployed staff from other clinics.	Staff from the organising clinic and deployed staff from other clinics.
	1.HFB staff	1.HFB staff	1.FMS	1.District health officer	1.FMS	1.FMS	1.FMS	1.FMS	1.FMS	1.FMS	1.FMS
	2.FMS	2.FMS	2.Doctors	2.FMS	2.Doctors	2.Doctors	2.Doctors	2.Doctors	2.Doctors	2.Doctors	2.Doctors
	3.Doctors	3.Doctors	3.SN	3.Doctors	3.SN	3.SN	3.SN	3.SN	3.SN	3.SN	3.SN
	4.SN	4.SN	4.MA	4.SN	4.MA	4.MA	4.MA	4.MA	4.MA	4.MA	4.MA
	5.MLT	5.MLT	5.MLT	5.MA	5.MLT	5.MLT	5.MLT	5.MLT	5.MLT	5.MLT	5.MLT
	6.Radiographer			6.MLT	6.Radiographer			6.Radiographer	6.Radiographer		
**Hajj health examination venue (separated / combined from outpatient service)**	Separated	Separated	Separated (from normal outpatient clinic which ran on Saturday)	Same waiting area with outpatient pool	Separated	Separated	Separated	Separated	Integrated with usual outpatient pool	Separated	Separated
**Hajj health examination process (separated / combined from outpatient service)**	Combined	Separated	Separated	Separated	Separated	Separated	Separated	Separated	Combined	Separated	Separated
	Indicated ‘Hajj pilgrims 2019’ at the printed queue number	Two registration tracks with one special track for senior citizens		Signs and checklist prepared for staff and patients’ easy referral	Special (separate) counters for (1) registration (2) laboratory tests, ECG, chest x-ray and (3) doctors consultation rooms for pilgrims	Two zones with same work process.	Three screening lines for (1) Vital signs/anthropometry (2) ECG, chest x-ray (3) Hb, random blood glucose, ABO grouping	Common registration counter.		Two tracks with same process. Each track consists of (1) Vital signs/anthropometry counter, (2) Five consultation rooms with one to two doctors for each room (3) One FMS	Screening room for (1) Vital signs/anthropometry (2) Check BRRJH if certain test is required
											After registration, patients put forms and BRRJH into a special box
**Stations**	1.Registration	1.Health screening questionnaire (for clinic key performance index)	1.Registration	1.Registration	1.Registration	1.Registration	1.Registration	1.Registration	1.Registration	1.At the entrance –completeness of documents checking	1.Registration
	2.Vital signs /anthropometry/document checking	2.Registration	2.Vital signs/anthropometry	2.HFB counter (for completeness of documents checking	2.Vital signs /anthropometry	2.HFB counter for document checking	2.Vital signs/ anthropometry	2.Vital signs/ anthropometry	2.Vital signs/ anthropometry	2.Registration	2.Vital signs/ anthropometry
	3. Laboratory tests, ECG, chest x-ray	3.Vital signs/ anthropometry	3.SSKM-20	3.Staff ran through BRRJH to check for special tests indication like ECG, chest x-ray	3.Laboratory tests, ECG, chest x-ray	3.Vital signs/ anthropometry	3.Laboratory tests, ECG, chest x-ray	3.Laboratory, ECG, chest x-ray	3.Completeness of documentation checking	3.Vital signs/ anthropometry	3.Laboratory tests, ECG, chest x-ray
	4.Doctor consultations	4.Laboratory tests, ECG, chest x-ray	4.Laboratory tests, ECG, chest x-ray	4.Vital signs/ anthropometry	4.Doctor consultations	4.ECAQ/PEFR counter	4.Pap smear for all married women above 40 y old	4.Consultation rooms	4. Laboratory, ECG, chest x-ray	4.Laboratory tests, ECG, chest x-ray	4.Doctor consultations
	5.Vaccination	5.Doctor consultations	5.Doctor consultations	5.Tests counter for random blood glucose, Hb, ABO grouping	5.Vaccination	5.Laboratory tests, ECG, chest x-ray	5.Doctor consultations	5.Vaccination	5.Consultation rooms	5.Doctor consultations	5.Vaccination
		6.Vaccination				6.Doctor consultation	6.Vaccination	6.Completeness of documentation checking by senior doctor.	6.Vaccination	6.Vaccination	6.HFB counter for completeness of documentation checking after examination.
						7.Vaccination					
**Available investigations**	ECG, chest x-ray, laboratory tests (glucose, ABO grouping, Hb, urinalysis)	1. ECG, chest x-ray, laboratory tests (glucose, ABO grouping, Hb, urinalysis)	ECG, chest x-ray, laboratory tests (glucose, ABO grouping, Hb, urinalysis)	ECG, chest x-ray, laboratory tests (glucose, ABO grouping, Hb, urinalysis)	ECG, chest x-ray, laboratory tests (glucose, ABO grouping, Hb, urinalysis)	ECG, chest x-ray, laboratory tests (glucose, ABO grouping, Hb, urinalysis)	1.ECG, chest x-ray laboratory tests (glucose, ABO grouping, Hb, urinalysis)	ECG, chest x-ray laboratory tests (glucose, ABO grouping, Hb, urinalysis)	ECG, chest x-ray laboratory tests (glucose, ABO grouping, Hb, urinalysis)	ECG, chest x-ray, laboratory tests (glucose, ABO grouping, Hb, urinalysis)	ECG, chest x-ray, laboratory tests (glucose, ABO grouping, Hb, urinalysis)
							2. Special test - pap smear for all married women above 40 y old				
**Referral/follow-up**	1. FMS	1. FMS	1. FMS	Referred mainly to FMS to resolve issues	Follow-up for review - patients given some time to optimise control	1. FMS	Referred mainly to FMS to resolve issues	1.FMS	Follow-up for review - patients were given some time to optimise	1.FMS	Referred mainly to FMS to resolve various issues
	2. Hospital specialists	2. Hospital specialists	2. Hospital specialists			2. Follow-up for review -patients were given some time to optimise control.		2.Follow-up for review - patients were given some time to optimise		2.Follow-up for review - patients were given some time to optimise	
	3. Follow-up for review - patients given some time to optimise control	3. Follow-up for review - patients given some time to optimise control	3. Follow-up for review - patients given some time to optimise control			3. To another clinic for long-term follow-up				3. To hospital for certain investigations	
	4. To hospital for certain investigations	4. To another clinic for long-term follow-up	4. To another clinic for long-term follow-up								
		5. To hospital for certain investigations like spirometry	5. To hospital for certain investigations								
**Continuity of care (COC)**	Follow-up for review whereby patients were given some time to optimise control.	No COC for most patients	Follow-up for review - patients given some time to optimise control	Referred to another clinic for long-term follow-up	Follow-up for review - patients given some time to optimise control	Referred to another clinic for long-term follow-up	No COC for some patients	Follow-up for review - patients given some time to optimise	Follow-up at the same clinic	No COC for many patients	No COC for many patients
		Referred to their clinics mostly for medications or investigations	No COC for some patients	No COC for some patients		No COC for some patients		Patients required individualised care were referred to their respective clinic		Referred to their clinics mostly for medications or investigations	Referred to their clinics mostly for medications or investigations
**Other remarks/special observations**	Some doctors were unaware of preliminary health screening form attached to the back of BRRJH and repeated certain investigations again	Doctors were briefed before the examination activities started	No previous medical records brought/traced from other centres	No previous medical records brought/traced from other centres	Few patients had records from other clinic	No previous medical records brought/traced from other centres	Preliminary health screening did not work well (blood tests results, specialist opinions and medications list unavailable)	Nil	No previous medical records brought/traced from other centres	Nil	Nil
		No previous medical records brought/ traced from other centres							Doctor did not know how to handle preliminary health screening form.		
		Documentations on both BRRJH and outpatient records (double entry)					Often preliminary health screening form not brought by patients				

SN – staff nurse, MA – medical assistant, MLT – medical laboratory technician, SSKM-20 - mental health status screening-20, Hb – haemoglobin, ECAQ - Elderly Cognitive Assessment Questionnaire, PEFR – peak expiratory flow rate

**Table 3:** Observation related to respiratory health and asthma care in public primary care clinics (n = 11)

	Clinic 1	Clinic 2	Clinic 3	Clinic 4	Clinic 5	Clinic 6	Clinic 7	Clinic 8	Clinic 9	Clinic 10	Clinic 11
**PFM/nomogram/spirometry**	No PFM	Children PFM (used for adults)	PFM available but not in all rooms	PFM available	PFM available	PFM available	PFM available	PFM available	PFM available	PFM available	PFM available
	No handheld spirometry	No nomogram	Nomogram (on the wall) available in all rooms	No nomogram,	No nomogram,	Nomogram, available	No nomogram,	No nomogram,	No nomogram,	No nomogram,	No nomogram,
		One handheld spirometry (but not used)	No handheld spirometry	No handheld spirometry	No handheld spirometry	No handheld spirometry	No handheld spirometry	No handheld spirometry	No handheld spirometry	No handheld spirometry	No handheld spirometry
		PEFR was performed by medical assistant in the treatment room by the doctor’s order.	PEFR was checked 3 times		PEFR were performed by doctor (technique was inconsistent and improper; no referral to nomogram)	One patient – PEFR done twice, one sitting and one standing	PEFR not done to all asthma patients			PEFR not done to all asthmatics example those with good control, not on MDI and children	Doctor referred to nomogram in the phone
					One doctor performed PEFR on most patients even without asthma	One patient – low PEFR reading was regarded as poor patient’s technique	Some patients PEFR done for two times only (no third reading)				
**Consultation** **(history-taking)**	Not applicable	Last attack, allergy, comorbidity	Onset of asthma, medications used, asthma attack for past week, exercise tolerance and triggers	Last attack, triggers, MDI use, history of admission	Symptoms, last attack, MDI use	Last attack, triggers, symptoms, MDI use	Last attack, MDI use, triggers	Symptoms, last attack, MDI use	Symptoms, last attack, MDI use	Last attack, MDI use, triggers	Last attack, medications compliance
		Doctors assessed asthma control by asking symptoms based on individual understanding and/or referred to the Hajj health examination and/or GINA guideline	Doctors assessed asthma control based on GINA guideline.	Lack of primary care consultation skills	Lack of primary care consultation skills		History relevant to asthma control was not taken properly by one medical officer who almost passed a patient with history of life-threatening asthma				
**Consultation** **(physical examination)**	Inconsistent and poor examination technique, auscultation over clothes	Adequate general and respiratory system examination	Inconsistent and poor examination technique, auscultation over clothes	Inconsistent and poor examination technique, auscultation over clothes	Inconsistent and poor examination technique, auscultation over clothes	Inconsistent and poor examination technique, auscultation over clothes	Poor examination technique, auscultation over clothes.	Inconsistent and poor examination technique, auscultation over clothes	Inconsistent and poor examination technique, auscultation over clothes, no attempt to lift head cover, lack of optimisation	Inconsistent and poor examination technique, auscultation over clothes	Inconsistent and poor examination technique, auscultation over clothes
						There was a doctor who did not examine one patient at all but passed the patient based on normal chest x-ray	Some doctor did no examination unless problem detected in the book but marked the examination as normal				
						No examination beds					
**Assessment and optimisation of control**	Lack of communication with patients, no motivation for patients for self- management and promotion	One asthma patient was sent for chest x-ray	Medications/MDI compliance and asthma control was not consistently emphasised.	No structured and inaccurate assessment of control on few patients	Poor communication, did not assess using GINA, focus on other chronic diagnosis care; lack of management of asthma, tend to refer to FMS for clinical decision	There was a patient referred to other clinics for long-term follow-up	Important asthma control points were not consistently assessed	Lack of optimisation of control	Lack of optimisation of control	MDI technique was not checked to all asthmatic patients (only for poorly controlled or if to refer to FMS).	No optimisation of a patient with poorly controlled asthma or referred to FMS but failed the patient
		One patient with uncontrolled asthma was referred to FMS, then referred for spirometry at hospital and given appointment for review.	Most patients with uncontrolled asthma were given some period of time for optimisation before were passed.	Some doctors were uncertain about management of uncontrolled asthma, did no step-up treatment and patients were referred to FMS after which step-up treatment was provided. This patient was referred for long-term follow-up at another clinic.		Poor clinical judgement (unsure if patient has COPD), did not check asthma control, lack optimisation, aim to certify only.	MDI technique was not checked	Care more for chronic disease		There was a patient referred for spirometry at hospital	
		Some doctors were uncertain about management of uncontrolled asthma, did no step-up treatment and patients were referred to FMS or other clinic for review.	Some doctors focused more on other chronic disease like hypertension and kidney-related for optimisation, control and reassessment.			One asthma patient – was informed of well-controlled asthma despite attack past 1 week, only can walk 1 flight of stairs and no further investigation was carried out	Few patients with uncontrolled asthma were referred for chest x-ray.			There was a practice of step-up asthma treatment.	
							Some patients’ control was optimised by step-up treatment while others were referred to FMS				
							More focused on other chronic disease like stroke				
**Preventive measures for attacks**	No specific advice on asthma	Some doctors were uncertain about frequency and intervals of vaccines; some doctors did not enquire about vaccination	Pilgrims advised not to forget to bring inhalers	No specific advice on asthma	No specific advice on asthma	No specific advice on asthma prevention given	Advised patients to wear mask during pilgrimage as dust might trigger asthma.	Some patients were informed about triggers at Saudi Arabia and advised to wear mask.	No specific advice on asthma	No specific advice on asthma	No specific advice on asthma
	Optional vaccinations (influenza, pneumococcal) were encouraged at private clinic		Optional vaccinations (influenza, pneumococcal) were encouraged at private clinic	Optional vaccinations (influenza, pneumococcal were encouraged at private clinic	Optional vaccinations (influenza, pneumococcal) were encouraged at private clinic	Optional vaccinations (influenza, pneumococcal) were encouraged at private clinic	Optional vaccinations (influenza, pneumococcal) were encouraged at private clinic	Optional vaccinations (influenza, pneumococcal) were encouraged at private clinic	Optional vaccinations (influenza, pneumococcal) were encouraged at private clinic	Optional vaccinations (influenza, pneumococcal) were encouraged at private clinic	Optional vaccinations (influenza, pneumococcal) were encouraged at private clinic

PFM – peak flow meter, PEFR – peak expiratory flow rate, GINA – Global Initiative for Asthma, MDI – metered dose inhaler, COPD – chronic obstructive pulmonary disease

The organisation of the Hajj health examination varied depending on a combination of factors including the number of pilgrims, individual clinic needs and resources. When the examination was carried out over the weekend, most of the clinic areas were utilised and the process appeared to be more systematic and better coordinated. Two clinics undertook Hajj health examinations on Saturday and ran their out-patient service concurrently in separate clinic areas and both ran smoothly. Some clinics that ran the examination on weekdays amidst their usual outpatient clinics used separate registration counters and queues, or provided special counters for vital signs and anthropometric measurements and for checking completeness of documents. Some clinics created multiple ‘stations’ for pilgrims to follow in a sequence which seemed to streamline the flow. One clinic provided a checklist to help pilgrims understand the work flow. One clinic that did not separate the Hajj health examination from the usual out-patient care had long queues and appeared chaotic. Clinics that provided staggered appointments for the Hajj health examination appeared to have a manageable process, despite conducting it during working days. For six clinics where we timed the process, the time from registration until the completion of the whole process ranged between 1.5 to 6 hours.

Vital signs (body temperature, blood pressure, pulse rate) anthropometric measurements (weight, height and body mass index), Malaysian mental health screening (SSKM-20) scale and the Elderly Cognitive Assessment Questionnaire (ECAQ) and investigations (random blood glucose, haemoglobin and ABO blood group) had to be completed in the BRRJH. Electrocardiogram (ECG) and chest x-ray (CXR) were arranged if indicated as per HFB guideline requirements [12]. At one clinic, Pap smear and pelvic examinations were done on female pilgrims who were married and aged 40 years and above and had consented. At another clinic, MOH health status screening questionnaires were administered to all pilgrims before undergoing the Hajj health examination. These two screenings were not part of the Hajj health examination requirements but were carried out as opportunistic screening in these two clinics.

The duration of doctor’s consultation timed at three clinics ranged between 15 to 30 minutes for each pilgrim. The assessments listed in the HFB checklist^12^ included a review of medical history, vital signs, physical examination, and laboratory tests when necessary. Breast examination is mandatory for all female pilgrims. All the findings, diagnosis and management plan were recorded in the BRRJH. Most of the consultation rooms at the public primary care clinics lacked privacy; with between 2 and 4 doctors carrying out consultations in the same room, making physical examination and maintenance of confidentiality impossible. We observed many doctors did not conduct a proper physical examination. For example, for examination of the respiratory system, auscultations were done over the clothes and only over two points.

Spirometry and nomogram for PEFR measurements were not readily available in most clinics. Handheld spirometry was available at one clinic, but it was not utilised. Most clinics assessed peak expiratory flow rate (PEFR) on pilgrims with asthma. However, the measurements were not done consistently for all patients. Variation was observed in the assessment of peak flow; this included the category of staff who conducted the PEFR, the technique and the interpretation of the measurements. Some clinics had staff nurses and medical assistants performing the PEFR prior to doctors’ consultations while others were conducted by the doctors as part of their consultations. Some clinics measured PEFR twice, some performed it on pilgrims without asthma, some used a paediatric peak flow meter for adults and some read the measured PEFR without referring to the nomogram [18].

The assessment of asthma control, medication adherence and inhaler techniques including spacer use or need were observed to be suboptimal. The interpretation of the PEFR readings did not seem to influence the doctor’s management. There seemed to be hesitation among the doctors on the assessment, management, and fitness certification for pilgrims with uncontrolled asthma. They typically referred pilgrims with uncontrolled asthma to a family medicine specialist for further management and fitness certification rather than optimising asthma treatment themselves. Pilgrims were mainly given general advice such as ensuring sufficient, non-expired medications including inhalers, to wear a mask to protect from dust and to stay hydrated. There was little/no individualised asthma education on preventive measures or provision of asthma action plans for the pilgrimage. Almost all patients were encouraged to get the optional influenza and pneumococcal vaccinations from private centres if they were willing to pay.

### Private primary care clinics

We observed two and three pilgrims who attended two private general practitioner (GP) clinics respectively for Hajj health examination. Pilgrims were encouraged to make appointments to ensure the availability of doctor who was eligible to certify the Hajj health examinations. Laboratory investigations were outsourced to private laboratories. The optional influenza and pneumococcal vaccines were readily available at both clinics. All pilgrims were charged a fee. With the very small number of pilgrims, the process of Hajj health examination at both clinics was manageable. Only one pilgrim with asthma attended one of the clinics. For this pilgrim, a thorough assessment of control and adequate respiratory examination were conducted. **Table 4** and **Table 5** summarise the overall finding of observations at the two private primary care clinics.

**Table 4:** Observations related to organisation of Hajj health examination in private primary care clinics (n = 2)

	General practitioner clinic 1	General practitioner clinic 2
**Screening period / day**	Any day during screening period. By appointment or walk-in	Any day during screening period. By appointment or walk-in
**Specific time**	No	No
**Organisation (centralised/decentralised)**	Not applicable	Not applicable
**No. of pilgrims**	Total three – two by appointments, 1 walked in	Total two – both walked in
	Pilgrim with asthma – one	Pilgrim with asthma – none
**Staff (same clinic/deployed from)**	One eligible doctor	Three eligible doctors
	Staff from the same clinic	Staff from the same clinic
**Place (separated/combined)**	Conducted in treatment room	Combined with outpatient pool
	Locum doctor runs the usual clinic	
**Process**	As part of normal clinic operation	As part of normal clinic operation
**Stations/Space**	No special stations	No special stations
**Investigations**	Available – Electrocardiogram, chest x-ray	Available – Electrocardiogram, chest x-ray
	Laboratory tests – Send to private laboratory	Laboratory tests – Send to private laboratory
	Few options of packages	Few options of packages
**Referral/follow-up**	Follow-up at the clinic	Referred to patients’ usual clinics mostly for medications/investigations
	No outside referral	
**Continuity of care**	None for most patients	None for most patients

**Table 5:** Observations related to respiratory health and asthma care in private primary care clinics (n = 2)

	General practitioner clinic 1	General practitioner clinic 2
**PFM/nomogram/spirometry**	PFM available	PFM available
	No nomogram	No nomogram
	No handheld spirometry	No handheld spirometry
	PEFR was done with correct technique (the 3 readings were not referred to nomogram)	
**Consultation (history-taking)**	Last attack, symptoms/fitness, medication/MDI use, follow-up, and allergy	Not applicable (no asthma pilgrims during observation)
**Consultation (physical examination)**	Respiratory examination: adequately done	Respiratory examination: adequately done
**Assessment and optimisation of control**	Optimisation of control: none, asthma control was good for both patients	Optimisation of control: not applicable
**Preventive measures for attacks**	General advice (to bring medications, check expiry, diet control and exercise)	General advice like prevent dehydration
	Optional vaccination available and encouraged (influenza, pneumococcal)	

PFM – peak flow meter, PEFR – peak expiratory flow rate, MDI – metered dose inhaler

### Outcomes of the Hajj health examination

There were four outcomes for pilgrims after the Hajj health examination: (i) passed and certified fit for Hajj, (ii) identified to have uncontrolled medical problem and referred for treatment optimisation followed by re-evaluation (iii) identified to have serious medical problem and referred to specialised disciplines at tertiary centres followed by re-evaluation, or (iv) failed and certified physically unfit for Hajj. All doctors performing the Hajj health examination were required to fill in a summary form in the BRRJH, stating the pilgrim’s final status of fitness certification for submission to the HFB database system.

## DISCUSSION

We observed considerable variation in the organisation of Hajj health examination among public primary care clinics. The implementation of centralised Hajj health examination posed a challenge to the health care providers in balancing the need for appropriate clinical evaluation and disease control, and the pressure for the Hajj certification within a limited time frame. As a result, Hajj health examination was mostly viewed as merely a ticket to certify fitness for pilgrimage rather than an opportunity to optimise chronic disease management including asthma. Poor physical examination of the respiratory system and suboptimal long-term management of chronic disease were two consistent observations related to asthma care and concerning aspects of the process that require further attention.

Two main disadvantages of the organisation of centralised clinic for Hajj health examination were time constraint due to heavy workload and unavailable medical records of pilgrims from external clinics. These compromised the comprehensiveness of clinical evaluation and disease management, provision of relevant health education including preventive measures and delayed the certification process for more complex cases. Time constraint is a recognised stress factor at work that can result in adverse consequences for primary care doctors and their patients’ care [19]. As the preliminary health assessment was not mandatory and was not often done, it was of concern that some pilgrims did not declare their known health issues. Moreover, unfamiliarity of the pilgrims to external doctors who were pooled to work at the centralised clinics possibly affected the establishment of appropriate doctor-patient relationship and hindered the delivery of health education and disrupted the continuity of care. Doctor-patient relationship is a powerful component of consultation and can alter various health related outcomes for patients. New patients, time-constraints and the health care setting are some identified factors that can adversely affect the doctor-patient relationship [20].

Despite time constraint being a contributing factor, the suboptimal assessment and management of asthma by the doctors, also significantly reflects the level of clinical competencies among primary care doctors, in particular the assessment of control, evaluation and management of uncontrolled asthma and provision of asthma education especially the asthma action plan. Many doctors did not seem to assess and manage the disease based on any guideline recommendation such as Malaysian Clinical Practice Guidelines (CPG) [18] or the GINA [21] recommendations. This is consistent with a study that found the implementation of asthma CPG as suboptimal in primary care [22]. Heavy workload and inadequate training were two important barriers to low adherence by primary care doctors to the guidelines [23]. Clinical pathway and supporting educational materials can be created and used to translate the evidence-based guidelines into succinct algorithms and facilitate the asthma care by primary care doctors [24,25]. Effective asthma self-management requires a comprehensive approach comprising of patients’ education and resources, professional skills and motivation and organisation priorities and routines [25].

The implementation of the Hajj health examination can be carried out more systematically if it is directed towards clearer objectives. The two main objectives should be (i) final certification of pilgrim’s health status and (ii) checking of the relevant documents. The HFB can make the Hajj preliminary health screening compulsory on receipt of the Hajj offer letter to ensure pilgrims’ health is assessed by their regular doctors or clinics in advance to the Hajj health examination. For asthma, this would provide ample time for optimisation of the pilgrims’ asthma control through adequate clinical evaluation including investigation and referral if necessary and opportunity to provide health education. Hence, the main role of doctors on duty for the Hajj health examination would be to consolidate the medical information prepared beforehand and establish the final status of fitness certification. For pilgrims with no known medical problem, the preliminary health screening is an avenue to detect occult disease and prompt earlier investigation, treatment, and disease stabilization. Neighbour country Indonesia uses an Integrated Hajj Computerized System (SISKOHAT), whereby pilgrims’ health information system is one of the integrated components [26]. It allows a longer and adequate time frame for pilgrims’ health optimisation and enables entry and update of pilgrims’ health information by all health services accessed by the pilgrims at any point of time. It involves three steps: screening at primary health care, disease control taking place in hospitals and final certification of fitness for Hajj. In step 1, their policy established a mandatory health screening and health coaching programs before the Hajj medical examination, as an effort to prepare fit and healthy pilgrims for Hajj. These programs comprise of a preliminary medical check-up, health promotion and prevention and physical exercise activities besides the Hajj rituals training [27,28].

For pilgrims, HFB could initiate and promote the importance of being fit and healthy for pilgrimage, by sending health reminders and guides to the pilgrims in the year before their Hajj scheduled year. This would hopefully trigger early awareness and efforts to achieve adequate disease control (including asthma) and sustain optimum health status to perform pilgrimage. Printed or electronic educational resources can provide quick access to information related to asthma. Supporting materials like videos or links to YouTube channels relevant to health and disease care can be provided along with the reminder [29].

This study is the first to observe the implementation and practice of the Hajj health examination in Malaysia with a focus on the delivery of care for pilgrims with asthma. It captured the real practice of Hajj health examination across a range of primary care organisations including the location, infrastructure and facilities, and the number of pilgrims. However, our selected clinics might not have included all organisational variations, and the days we observed might not have been typical while the number of private general practitioner was too small to draw definitive conclusions on the practice of Hajj health examination at private primary care clinics. Moreover, participation in research and the presence of an observer might have affected the health care providers’ behaviour and clinical conduct, especially of asthma care. Studies on the views of pilgrims and various stakeholders involved in the Hajj health examination process should provide more supportive data. Nevertheless, the findings may provide performance data for analysis and basis for potential avenues to improvement by the HFB and MOH. Besides asthma, it could also be extended to other chronic diseases to influence future strategies to improve care among pilgrims.

## CONCLUSION

As a conclusion, there is great potential to reform the Hajj health certification process in Malaysia and to improve the provision of asthma care in primary health care. Strategies to restructuring the delivery of Hajj health examination could address time constraint, clinical competency of primary health care providers and resources limitations. This is to reduce the risks posed from asthma, and by extension the other chronic diseases, not only during pilgrimage but also on the impact to the long-term health.
